# Enteric Viral Infections among Domesticated South American Camelids: First Detection of Mammalian Orthoreovirus in Camelids

**DOI:** 10.3390/ani11051455

**Published:** 2021-05-19

**Authors:** Dayana Castilla, Victor Escobar, Sergio Ynga, Luis Llanco, Alberto Manchego, César Lázaro, Dennis Navarro, Norma Santos, Miguel Rojas

**Affiliations:** 1 Laboratorio de Inmunología, Facultad de Medicina Veterinaria, Universidad Nacional Mayor de San Marcos, Lima, Apartado 03-5137, Peru; dayana.castilla@unmsm.edu.pe (D.C.); victor.escobar@unmsm.edu.pe (V.E.); sergio.ynga@unmsm.edu.pe (S.Y.); amanchegos@unmsm.edu.pe (A.M.); 2 Laboratorio de Zootecnia y Producción Agropecuaria, Facultad de Medicina Veterinaria, Universidad Nacional Mayor de San Marcos, Lima, Apartado 03-5137, Peru; lllancoa@unmsm.edu.pe; 3 Laboratorio de Farmacología y Toxicología Veterinaria, Facultad de Medicina Veterinaria, Universidad Nacional Mayor de San Marcos, Lima, Apartado 03-5137, Peru; clazarod@unmsm.edu.pe; 4 Laboratorio de Virología, Facultad de Medicina Veterinaria, Universidad Nacional Mayor de San Marcos, Lima, Apartado 03-5137, Peru; dnavarrom@unmsm.edu.pe; 5 Instituto de Microbiologia Paulo de Góes, Universidade Federal do Rio de Janeiro, Rio de Janeiro 21941-902, RJ, Brazil; nsantos@micro.ufrj.br

**Keywords:** alpacas, llamas, Peru, rotavirus, coronavirus, mammalian orthoreovirus

## Abstract

**Simple Summary:**

South American camelids (SACs) constitute the greatest livestock wealth of the Andean populations. Approximately half a million people from the high Andean areas are dedicated to the breeding of SACs as their main activity. In general, infectious diseases, particularly diarrheal infections, cause high morbidity and mortality in offspring and adult animals. In the study, we demonstrated that multiple virus pathogens circulate among neonatal SACs, and coinfections from other viruses might be common among SAC crias. We also demonstrated, for the first-time anywhere, the circulation of mammalian orthoreovirus in SACs or camelids. Diarrheal infections can potentially impact livestock productivity, which translates into serious economic losses for the Peruvian SAC industry, especially within rural communities, directly impacting their livelihood. Better knowledge of the infections that afflict these animals will enable the implementation of measures for the prevention and control of pathogens, the results of which will ultimately be reflected in improving the quality of life of these communities.

**Abstract:**

Enteric infections are a major cause of neonatal death in South American camelids (SACs). The aim of this study was to determine the prevalence of enteric viral pathogens among alpacas and llamas in Canchis, Cuzco, located in the southern Peruvian highland. Fecal samples were obtained from 80 neonatal alpacas and llamas and tested for coronavirus (CoV), mammalian orthoreovirus (MRV), and rotavirus A (RVA) by RT-PCR. Of the 80 fecal samples analyzed, 76 (95%) were positive for at least one of the viruses tested. Overall, the frequencies of positive samples were 94.1% and 100% among alpacas and llamas, respectively. Of the positive samples, 33 (43.4%) were monoinfected, while 43 (56.6%) had coinfections with two (83.7%) or three (16.3%) viruses. CoV was the most commonly detected virus (87.5%) followed by MRV (50%). RVA was detected only in coinfections. To our knowledge, this is the first description of MRV circulation in SACs or camelids anywhere. These data show that multiple viruses circulate widely among young alpaca and llama crias within the studied areas. These infections can potentially reduce livestock productivity, which translates into serious economic losses for rural communities, directly impacting their livelihoods.

## 1. Introduction

South American camelids (SACs) constitute the greatest livestock wealth of Andean populations. The raising of alpacas and llamas is a crucial economic activity in Andean regions, enabling the production of fibers; primarily those of alpacas, which have a high value in international markets because of their fine texture. The llama, due to its size and physical strength, is also used as a pack animal and plays an important role in rural transportation. Peru possesses three million alpacas, the largest alpaca population in the world, and has a herd of approximately one million llamas. Most of these animals are found in the departments of the southern highlands, particularly in Puno and Cuzco. Over 80% of Peru’s alpacas and the entire population of llamas are kept by small producers who lack adequate infrastructure for conducting both production and marketing activities of their products [1,2,3]. According to the Peruvian Ministry of Agrarian Development and Irrigation [1], Cuzco is the second most important center for alpaca and llama breeding in Peru, with the province of Canchis being the main producer. Unfortunately, neonatal mortality rates among alpacas and llamas in this province are 30% and 25%, respectively [1]. Diseases cause great losses due to both mortality and decreased productivity. Enteropathies are a major cause of neonatal death in SACs. The most significant pathogens causing diarrhea in SACs are Coccidia, *Cryptosporidium* spp., *Giardia* spp., *Salmonella* spp., *Escherichia coli*, *Clostridium* spp., rotavirus, and coronavirus [4,5,6,7,8,9,10,11]. 

Despite the vital importance of alpaca and llama breeding to the Peruvian economy, studies on enteric infections, particularly viral infections, in SACs are incipient. A better understanding of the epidemiology of enteric infections is essential to develop preventive measures. The aim of this study was to determine the prevalence of enteric viral pathogens—specifically coronavirus (CoV), mammalian orthoreovirus (MRV), and rotavirus A (RVA)—associated with infections among alpacas and llamas in Canchis, Cuzco, located in the southern Peruvian highland.

CoV and RVA have already been identified as important diarrheal pathogens in neonatal SACs. On the other hand, MRV has not been associated to enteric infection. However, in a previous study, during the characterization of RVA strains detected in feces of alpacas and llamas collected in February 2014 in the province of Canchis in the state of Cusco, Peru, three viral strains containing 10 segments of double-stranded RNA (dsRNA) suggestive of MRV were isolated in an MA-104 cell culture [12]. In this study, we describe the characterization of these strains and investigate the prevalence of this virus in these animals.

## 2. Materials and Methods

### 2.1. MRV Isolation and Characterization

The protocol used for isolating MRV in cultures of African green monkey cells (MA-104) has been described elsewhere [12]. Viral culture supernatants were examined using polyacrylamide gel electrophoresis (PAGE) followed by silver nitrate staining [13]. Based on the PAGE profile suggestive of the MRV genome, specific primers were designed and used for amplification and sequencing of the MRV sigma 1 gene (Supplementary Table S1). The amplified genomic segments were sent for sequencing to Macrogen Inc. (Seoul, Korea). Overlapping sequences were assembled and edited using SeqMan, EditSeq, and MegAlign in the Lasergene software package (DNASTAR, Madison, WI, USA). Phylogenetic analysis was performed with MEGA software (version 7.0.14). Dendrograms were constructed using the maximum likelihood method based on the GTR-G model. Statistical significance was estimated by bootstrap analysis with 1000 pseudoreplicates. The sequences were compared to reference MRV strains obtained from GenBank (https://www.ncbi.nlm.nih.gov/nucleotide/, accessed on 5 April 2021). Sequences generated in this study were deposited in GenBank under the accession numbers MN200219, MN200220, and MN200221.

### 2.2. Fecal Samples

Eighty fecal samples were collected from neonatal alpacas (*n* = 68) and llamas (*n* = 12), all one to five weeks old, with (*n* = 43; 35 alpacas and 8 llamas) and without (*n* = 37; 33 alpacas and 4 llamas) diarrhea, from three camelid breeding areas of the districts of Marangani (Silly and Quisini) and Sicuani (Pataccalasaya), province of Canchis, department of Cuzco, Peru. Samples were collected during the birthing season of alpacas and llamas, in January and February 2015. Fecal samples were collected directly from the rectum of animals and kept at −20 °C until processing at the Laboratory of Veterinary Virology and Immunology, Universidad Nacional Mayor de San Marcos, Lima, Peru.

### 2.3. Viral RNA Extraction

The extraction of viral RNA samples required an initial clarification process. Fecal suspensions were prepared at 10–20% in PBS (pH 7.2) and then centrifuged at 2500× *g* for 5 min. Supernatant was filtered using a 0.22 μM diameter filter. Viral RNA was extracted from clarified fecal supernatants using TRIzol™ LS Reagent (Thermo Fisher Scientific, Waltham, MA, USA) according to the manufacturer’s recommendations.

### 2.4. Viral Detection and Identification

Samples were submitted to RT-PCR and nested PCR using specific primers (Table 1). Reverse transcription polymerase chain reaction (RT-PCR) and nested PCR were performed using the GoScript™ reverse transcription system and GoTaq^®^ Green Master Mix (Promega, Madison, WI, USA), respectively. The genomic RNAs were subject to one cycle of reverse transcription (5 min at 25 °C followed by 45 min at 42 °C), and one step of 2 min at 95 °C followed the cycles of PCR. 

For CoV testing, the primers Cor-FW and Cor-RV, which amplify a 251-bp fragment of the RNA-dependent RNA polymerase (RdRp) gene of any CoV, were used for the first round of PCR, according to the protocol described previously [14]. Five microliters of the amplicons produced in the first round of PCR were used for BetaCoV detection, using the primers Beta.CoV.F and Beta.CoV.R, to generate a 227-bp fragment of polymerase-encoding gene. The PCR conditions were as follows: 3 min at 95 °C; followed by 35 cycles of PCR, each consisting of 40 s at 94 °C, 1 min at 57 °C, and 40 s at 72 °C; and the final extension step for 10 min at 72 °C. BetaCoV-positive samples were subsequently analyzed by nested PCR for the identification of the subgenus *Embecovirus* (formerly known as BetaCoV lineage A, BetaCoV 1, or bovine-like CoV (BCoV-like)), using the first-round PCR products and specific CV2U and CV2L primers [15] that amplify all Embecovirus strains except for HCoV-HKU1 (AY597011) and ChRCoV (KM349742), with a predicted product of 136 bp. The PCR conditions were the same as described above for BetaCoV. 

MRV detection was performed by one round of RT-PCR using the primers MRV-FM and MRV-RM. The primers used for MRV RT-PCR detection target the conserved genomic segment that encodes the RdRp. Selection was carried out in silico by aligning all MRV genomes deposited on GenBank, representatives of all serotypes (MRV1-MRV4), with a predicted product of 181 bp. The specificity of the primers was tested by amplifying the cell-cultured MRV strains and confirming the results by sequencing analysis. Sequences generated from the RdRp 181-bp amplicons were also deposited in GenBank under the accession numbers MN200216, MN200217, and MN200218. The PCR conditions were as follows: 3 min at 95 °C; followed by 40 cycles of PCR, each consisting of 40 s at 94 °C, 1 min at 45 °C, and 1 min at 72 °C; and the final extension step for 5 min at 72 °C. 

RVA was detected using primers that amplify the NSP5-encoding gene, as described previously [12,16]. 

All primers were synthesized by IDT (Integrated DNA Technologies, Coralville, IA, USA). Reactions were performed in a Bio-Rad T100 PCR thermal cycler (Bio-Rad Laboratories, Hercules, CA, USA). The PCR products were separated by 1.5% (*w/v*) agarose gel electrophoresis, stained with ethidium bromide, and visualized under UV light. A 100-bp DNA ladder (Promega, Madison, WI, USA) was used to determine molecular size.

To validate the PCR assays, positive controls were used for each of the studied viruses, which consisted of viral strains isolated in cell culture whose identification was previously confirmed by sequencing analysis. Strains AlpCoV-SA44 and AlpCoV-HN (GenBank accession numbers KX266949 and KX266944, respectively), both BetaCoV, subgenus *Embecovirus*, were used as CoV-positive controls. For MRV, strains SA44-Alpaca, H8-Alpaca, and SL7-Llama (GenBank accession numbers MN200216, MN200217, and MN200218, respectively), identified as MRV-1, were used. RV strains RVA/Alpaca-tc/Per/ SA44/2014/G3P(30) (GenBank accession number KT935485) and RVA/Human-wt/Per/FRNM/2014/G3P(8)(14)(40)(50) (GenBank accession number KY972105), belonging to rotavirus species A (RVA), were used as positive controls.

## 3. Results

### 3.1. MRV Characterization by PAGE and Sequencing

**Figure 1:**
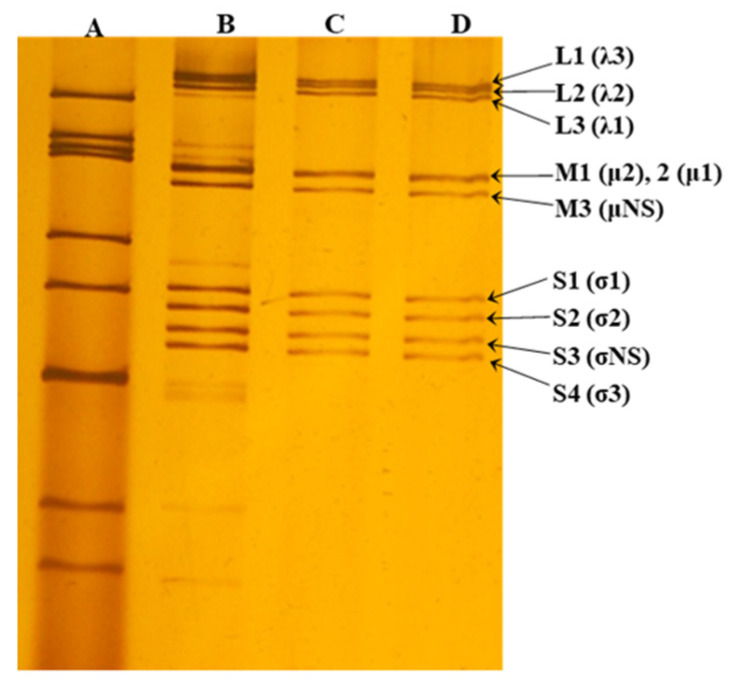
Polyacrylamide gel electrophoresis (PAGE) analysis of MRV dsRNA. PAGE of three strains isolated from intestinal contents of two alpacas (SA44 and H8) and one llama (SL7) in MA-104 cell line showing the 10 characteristic genomic segments of MRV: (A) SA-11 RVA strain exhibiting the typical 4–2–3–2 pattern (11 segments of dsRNA); (B) SA44 strain isolated showing a coinfection of RVA (11 genomic segments) and MRV (10 genomic segments); (C and D) strains H8 and SL7 exhibiting MRV monoinfection, showing the 10 dsRNA segments characteristic of MRV. Each segment of dsRNA is classified by size: genes L1–L3 (large segments), M1–M3 (medium segments) and S1–S4 (short segments) encode the proteins λ3–λ1, μ2-μNS, and σ1–σ3, respectively.

**Figure 2:**
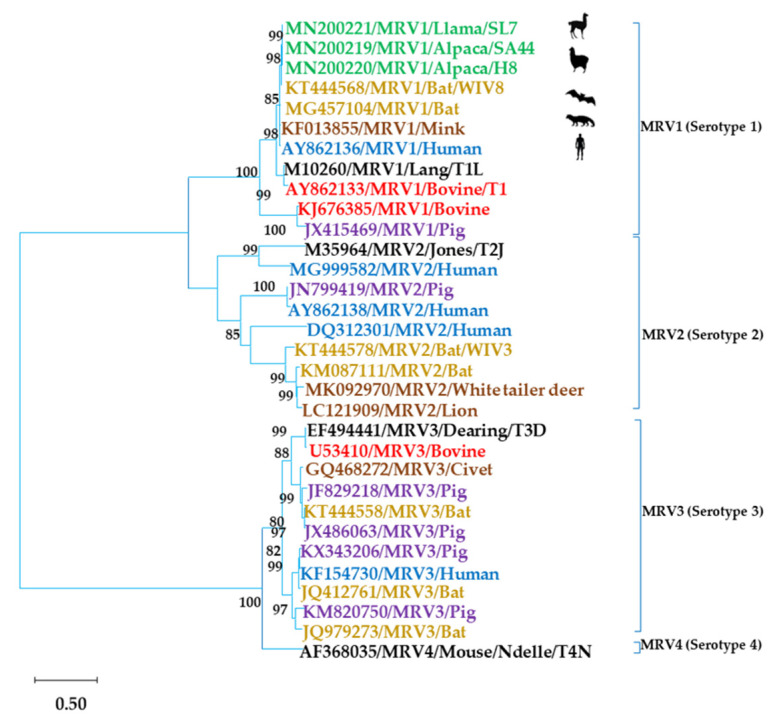
Phylogenetic trees constructed from partial nucleotide sequences (1069 bp) of the sigma 1 gene from MRV strains. Distances were corrected with the GTR-G model. Phylogenetic trees were constructed using the maximum likelihood method. Statistical support was provided by bootstrapping 1000 pseudoreplicates. Bootstrap values > 75% are given at branch nodes. The distance scale reflects substitutions/site. Reference samples are identified by GenBank accession numbers. In black, reference strains for each serotype.

Analysis of the migration profile of the dsRNA genome of three viral strains (SA44, H8, and SL7) isolated in MA-104 cells revealed an electrophoretic migration profile compatible with MRV (Figure 1). A sequence of 1069 bp of the viral attachment protein sigma 1-encoding gene was obtained for the alpaca (SA44 and H8) and llama (SL7) strains. They showed a nucleotide identity of 96.7% to 99.3% with bat strains, 96.7% to 96.9% with human strains, and 96.5% with mink MRV strains. Phylogenetic analysis identified those strains as serotype 1 (MRV1) (Figure 2).

### 3.2. Virus Detection

Of the 80 fecal samples, 76 (95%) were positive for at least one of the screened viruses. Overall, the frequencies of positive samples were 94.1% and 100% among alpacas and llamas, respectively (Table 2). Positive samples were detected in all three sites of collection. Viral infections were observed among both symptomatic and asymptomatic animals, with no statistically significant difference (*p* = 0.1048). Specifically, among the 68 samples from alpacas, 26 were from animals with diarrhea and all tested positive. Of the 42 samples from alpacas without diarrhea, 38 tested positive. Among the 12 samples from llamas, 9 and 3 were from animals with and without diarrhea, respectively, and all tested positive.

Of the positive samples, 33 (43.4%) were monoinfected, while 43 (56.6%) had coinfections of two or three viruses (Table 2). The community with the highest rate of coinfections was Silly, at 69.7% (23/33) among alpacas and 41.7% (5/12) among llamas, followed by Quisini and Pataccalasaya, at 47.8% (11/23) and 33.7% (4/12) among alpacas, respectively (Table 2). CoV was the most commonly detected virus in all studied communities, being present in 95.3% (*n* = 29) of monoinfections and 95.3% (*n* = 41) of coinfections. MRV was found in 12.1% (*n* = 4) of monoinfections and 83.7% (*n* = 36) of coinfections. RVA was only found in coinfections (37.7%; *n* = 16) (Table 2).

### 3.3. Identification of CoV Genus and Subgenus

Of the 70 samples positive for CoV, 66 (94.3%) were identified as BetaCoV. Of these, 16 (22.9%) belonged to the subgenus *Embecovirus*, with the majority of these samples (15/16) obtained from alpacas. The genus was not identified for 4 (5.7%) and 71.4% of CoV and BetaCoV samples, respectively (Table 3).

## 4. Discussion

There are few reports on enteric viral infections in SACs, particularly in Peru [6,8,9,10,17,18]. The majority of published studies were conducted in research centers with semi-intensive production systems with good nutritional and health management. Our study focused on small producers in rural communities with extensive production systems and inadequate health management training, who are responsible for more than 80% of the SAC herd in Peru. 

In Peru, the SAC breeding area is geographically located over 3800 m above sea level. This presents an extreme climate and poor forage for animal species that are not typical of the Andean plateau, although it is possible to observe the rare presence of traditional livestock (cattle, sheep, horses, and pigs). An estimated one and a half million people from the high Andean areas were engaged in the breeding of SACs as their primary economic activity in the year 2000. However, the per capita income in these camelid-producing areas is the lowest in the country [3,19,20].

The economic conditions of peasant communities such as those studied here, which are dedicated to the breeding of SACs, are chaotic due to extreme poverty. Their situation is becoming even more critical due to the lack of drinking water, vital hygienic services, and healthcare. These shortages are reflected in the health of their animals, which suffer greater disease burdens due to inadequate handling and lack of prophylactic programs. The animals are managed in extensive systems that use swampy areas watered by streams or rivers. Infectious and parasitic diseases are major limiting factors in the production of these animals. In general, infectious diseases cause high morbidity and mortality in both offspring and adult animals, which translate into serious economic losses for rural communities, directly impacting their livelihood [4,6,21].

Our data showed a wide circulation of the surveyed viruses among alpacas and llamas in the three studied communities. CoV and RVA have already been identified as important diarrheal pathogens in neonatal SACs [6,10,17,22]. MRV, on the other hand, has been associated primarily with mild respiratory and enteric infections in mammals. Yet in the last decade, MRV has been associated with upper respiratory tract infections, encephalitis, and diarrhea [23,24,25,26,27,28,29]. In a previous study, we accidentally isolated three viral strains whose genome presented an electrophoretic profile similar to MRV [12]. The characterization of these isolates confirmed their identification as MRV1, with a high nucleotide identity with MRV1 strains from bats, humans, and mink (Figure 2). Therefore, we investigated the presence of MRV in new fecal samples of alpacas and llamas in an attempt to determine whether the detection of these strains was a rare event or whether this virus circulated frequently among these animals. In this study, MRV was the second most prevalent virus, detected in 50% of samples. In the Americas, this virus has been associated with diarrhea in calf and deer in the USA [24,27] and in humans in Brazil [30]. To our knowledge, this is the first description of MRV circulation in SACs or camelids anywhere. The high prevalence of infected animals suggests that this virus is well adapted to the environmental conditions of the Peruvian Andean highlands and local SAC herds.

Previous studies conducted in the Andean areas of the Peruvian highlands have shown the circulation of CoV in alpacas on farms with semi-intensive systems and in communities with extensive systems [6,8,22], with infection rates of 18.3% to 53.3%. On the other hand, in alpacas reared under semi-intensive systems in research centers, CoV was detected in 26.8% of animals [17]. The variation in rates observed in different studies probably reflects differences in methodologies, the breeding system employed, and the sanitary conditions of each location. However, it is clear that this virus is widespread in Peruvian alpaca herds. Studies on CoV in llamas have not been reported in Peru. However, previous studies have disclosed CoV infections in outbreaks of diarrhea in llamas in the USA [7,21]. We observed a CoV infection rate of 91.7% among these animals. 

Nucleotide sequences of alpaca CoV strains exhibited high identity with circulating bovine strains (EmbeCoV, formerly BetaCov 1), suggesting a possible bovine origin of these viruses [10,16,30,31]. The majority of CoV strains detected in alpacas (72.7%) and llamas (90.1%), although belonging to the *Betacoronavirus* genus, do not belong to the *Embecovirus* subgenus. These data suggest the circulation of different genera and subgenera of CoV among these animals.

RVA has already been associated with outbreaks of diarrhea among alpacas and llamas in the Peruvian highlands with high mortality rates [6,9,22,32]. Interestingly, in this study, RVA was only detected in coinfections. High rates of coinfections involving CoV and RVA have been described among SACs previously [6,22].

Virus-positive samples were detected among animals with and without diarrhea. The occurrence of asymptomatic infections in these animals could be due to the persistence of maternal immunity, or to the fact that as these pathogens are circulating widely in the herd, animals could have been infected before the study period and may have developed immunity, thus not presenting with diarrhea. Asymptomatic shedding is a source of contamination favoring the environmental persistence of these pathogens.

One of the most impressive findings of this study was the extremely high prevalence of coinfections (56.6%). This can be explained by (i) the high sensitivity of the detection technique used in the study, (ii) the high circulation of these viruses among herds, and (iii) inadequate sanitary conditions for managing the animals, allowing the dissemination of pathogens. Unfortunately, we do not have information that allows us to infer the impact of coinfections on clinical presentations. However, it has been demonstrated that coinfections probably increase the severity of diarrhea in alpacas [6].

## 5. Conclusions

The raising of alpacas and llamas in Peru is conducted in a particular ecological niche by rural communities and is characterized by the close contact of shepherds with animals as well as failures in sanitary and prophylactic programs due to lack of economic resources. These factors facilitate interspecies transmission of viruses such as CoV, MRV, and RVA, triggering possible zoonotic or anthropozoonotic infections [32,33,34]. Consequently, epidemiological surveillance is essential to prevent and control the emergence or re-emergence of new viral genotypes and variants with zoonotic potential.

## Figures and Tables

**Table 1:** Primers used in the RT-PCR and nested PCR assay for CoV, MRV, and RVA detection ^†^.

Virus	Gene	Assay	Primer *	Primer Sequence 5′→3′	Position	Product Size (bp)	Reference
All CoV	RpRd ^+^	RT-PCR	Cor-FW	ACWCARHTVAAYYTNAARTAYGC	14,922–14,944	251	[14]
			Cor-RV	TCRCAYTTDGGRTARTCCCA	15,153–15,172		
BetaCoV	RpRd	Nested PCR	Beta.CoV.F	ATTAGTGCWAAGAATAGAGCYCGCAC	14,946–14,971	227	This study
			Beta.CoV.R	TCACAYTTWGGRTARTCCCADCCCA	15,148–15,172		
*Embecovirus*	RpRd	Nested PCR	CV2U.F	TACTATGACTGGCAGAATGTTTCA	14,996–15,019	136	[15]
			CV2L.R	AACATCTTTAATAAGGCGRCGTAA	15,108–15,131		
All MRV	RpRd	RT-PCR	MRV-FM	CCNATATCNGGAATGCAGAA	1943–1962	181	This study
			MRV-RM	TCCATCATCGTRCTATTRTTNGC	2102–2124		
All RVA	NSP5	RT-PCR	Gen_NSP5F	GGCTTTTAAAGCGCTACAG	1–19	667	[16]
			Gen_NSP5R	GGTCACAAAACGGGAGT	651–667		
All RVA	NSP5	Nested PCR	Max_1FM	CGTCAACTCTTTCTGGAAAATCTA	95–121	562	[12]
			Max_4RM	GTGGGGAGCTCCCTAGT	637–656		

^†^ CoV = coronavirus; MRV = mammalian orthoreovirus; RVA = rotavirus A. ^+^ RdRp = RNA-dependent RNA polymerase. * Primers’ positions were determined based on the reference CoV strain DQ915164, the MRV strain M24734, and the RVA strain KT935485.

**Table 2:** Distribution of viral infections among alpacas and llamas in three rural communities of the province of Canchis, Peru.

Community/District	Geographic Coordinates	Host Species	N^o^ Tested Samples	N^o^ of Positive Samples (%)				
				CoV	MRV	RVA	Coinfections ^†^	Total
Silly/Marangani	14°21′12″ S, 71°10′17″ W, 3800 masl ^±^	Alpaca	33	7	2	0	23 (16 CoV + MRV; 3 CoV + RVA; 1 MRV + RVA; 3 CoV + MRV + RVA)	32 (97)
Quisini/Marangani	14°39′72″ S, 71°10′89″ W, 4300 masl	Alpaca	23	10	0	0	11 (4 CoV + MRV; 3 CoV + RVA; 4 CoV + MRV + RVA)	21 (91.3)
Pataccalasaya/Sicuani	14°16′9.5″ S, 71°9′39.1″ W, 4700 masl	Alpaca	12	6	1	0	4 (2 CoV + MRV; 1 CoV + RVA; 1 MRV + RVA)	11 (91.7)
Total			68	23	3	0	38	64 (94.1)
Silly/Marangani	14°21′12″ S, 71°10′17″ W, 3800 masl ^±^	Llama	12	6	1	0	5 (CoV + MRV)	12 (100)
Total			80	29	4	0	43	76 (95)

CoV = coronavirus; MRV = mammalian orthoreovirus; RVA = rotavirus species *A*. ^†^ Coinfection with two or three viruses. ^±^ meter above sea level.

**Table 3:** Identification of genus *Betacoronavirus* and subgenus *Embecovirus* in alpacas and llamas.

Host Species	N^o^ of Samples	Genus/Subgenus		
		BetaCoV/EmbeCoV	BetaCoV/ Not Identified	Genus/Subgenus Not Identified
Alpacas	59	15	40	4
Llamas	11	1	10	0
Total	70	16 (22.9%)	50 (71.4%)	4 (5.7%)

BetaCoV = genus *Betacoronavirus*; EmbeCoV = subgenus *Embecovirus*.

## Data Availability

The data presented in this study are available in Supplementary material and in GenBank database (https://www.ncbi.nlm.nih.gov/genbank/, accessed on 5 April 2021) (accession numbers MN200216-MN200221).

## Supplementary Materials

The following are available online at https://www.mdpi.com/article/10.3390/ani11051455/s1, Table S1: Primers used for genotyping (serotype) of MRV1, MRV2, MRV3 and MRV4 of the controls isolated in the MA104 cell line.
