# Supplementary material for: Enteric Viral Infections among Domesticated South American Camelids: First Detection of Mammalian Orthoreovirus in Camelids
Source: Animals (Basel). 2021 May 19;11(5):1455. doi: 10.3390/ani11051455 (PMC8159077; doi:10.3390/ani11051455)
Supplement: Supplementary file 1 [file animals-11-01455-s001.zip › animals-1197783-supplementary.pdf]

Table S1. Primers used for genotyping (serotype) of MRV1, MRV2, MRV3 and MRV4 of the controls isolated in the MA104 cell line.

**Virus**	**Gene**	**Assay**	**Primer***	**Primer sequence 5′→3′**	**Position**	**Product size (pb)**	**Reference**
MRV1	S1 (σ1) ⁺	RT-PCR	MRV1-F1	GCTATTCGCGCCTATGGATGC	1-21	1421	This study
			MRV1-R1	CTCACATTRCAAGGATACAT-KATCGTC	1395-1421		
MRV2	S1 (σ1) ⁺	RT-PCR	MRV2-F2	GCTATTCGYACTSATGTC	2-19	1389	This study
			MRV2-R2	TACATMATNGTCATCGGC	1373-1390		
MRV3-4	S1 (σ1) ⁺	RT-PCR	MRV3-4.F	GCTATTKGTCGKATGGAT	3-20	1394	This study
			MRV3-4.R	GRTCCTCACGTGAARCT	1379-1396		

MRV1 = mammalian orthoreovirus serotype 1; MRV2 = mammalian orthoreovirus serotype 2; MRV3-4 = mammalian orthoreovirus serotype 3 and 4. ⁺S1 = Sigma-1. * Primers position were determined based on reference MRV1 – T1 Lang strain M14779, the MRV2-T2 strain M35964 and MRV3-4 – T3 strains EF494441.
